# The relationship between capacity and utilization of nonpharmacologic therapies in the US Military Health System

**DOI:** 10.1186/s12913-022-07700-4

**Published:** 2022-03-07

**Authors:** Rendelle Bolton, Grant Ritter, Krista Highland, Mary Jo Larson

**Affiliations:** 1 The Heller School for Social Policy and Management, Brandeis University, 415 South Street, MA 02453 Waltham, USA; 2 US Department of Veterans Affairs, VA Bedford Healthcare System, Center for Healthcare Organization and Implementation Research, 200 Springs Road, Bedford, MA 01730 USA; 3 Department of Anesthesiology, Defense and Veterans Center for Integrative Pain Management, Uniformed Services University, 11300 Rockville Pike, Suite 709, Rockville, MD 20852 USA; 4 Henry M. Jackson Foundation, 11300 Rockville Pike, Suite 709, Rockville, MD 20852 USA

**Keywords:** Nonpharmacologic therapies, Complementary and integrative health, Military, Access, Healthcare utilization, Professional capacity, Pain therapy, Physical therapy

## Abstract

**Background:**

Nonpharmacologic therapies (NPTs) are recommended as first-line treatments for pain, however the impact of expanding professional capacity to deliver these therapies on use has not been extensively studied. We sought to examine whether an effort by the US Military Health System (MHS) to improve access to NPTs by expanding professional capacity increased NPT utilization in a cohort at higher risk for pain – Army soldiers returning from deployment.

**Methods:**

Our study involved secondary analysis of MHS workforce data derived from the Defense Medical Human Resources System Internet (DMHRSi), and healthcare utilization data obtained from two ambulatory record systems of the Military Health System (MHS) for a sample of 863,855 Army soldiers previously deployed to Iraq or Afghanistan over a 10-year period (2008–2017). We measured clinical provider capacity in three occupational groups responsible for pain management at 130 military treatment facilities (MTFs): physical therapy, chiropractic, and behavioral health, measured annually as full-time equivalence per 100,000 patients served at each MTF. Utilization in both direct and purchased care settings was measured as annual mean NPT users per 1000 sample members and mean encounters per NPT user. Generalized estimating equation models estimated the associations of facility-level occupational capacity measures and facility-level utilization NPT measures.

**Results:**

In 2008, nearly all MTFs had some physical therapist and behavioral health provider capacity, but less than half had any chiropractor capacity. The largest increase in capacity from 2008 to 2017 was for chiropractors (89%) followed by behavioral health providers (77%) and physical therapists (37%). Models indicated that increased capacity of physical therapists and chiropractors were associated with significantly increased utilization of six out of seven NPTs. Acupuncture initiation was associated with capacity increases in each occupation. Increased professional capacity in MTFs was associated with limited but positive effects on NPT utilization in purchased care.

**Conclusions:**

Increasing occupational capacity in three professions responsible for delivering NPTs at MTFs were associated with growing utilization of seven NPTs in this Army sample. Despite increasing capacity in MTFs, some positive associations between MTF capacity and purchased care utilization suggest an unmet need for NPTs. Future research should examine if these changes lead to greater receipt of guideline-concordant pain management.

**Supplementary Information:**

The online version contains supplementary material available at 10.1186/s12913-022-07700-4.

## Background

Patients and healthcare systems alike are increasingly turning to nonpharmacologic therapies (NPTs) to address multiple health conditions including acute and chronic pain [1–3]. These approaches include physical therapy, chiropractic care, cognitive behavioral therapy, mindfulness, and acupuncture [4]. Reflecting a growing body of evidence on their acceptability, potential effectiveness for pain [5, 6], as first line treatment to reduce risk from high dose or long-term opioids [7, 8], and to mitigate or delay surgery and other high cost procedures [9, 10], NPTs are now incorporated as part of comprehensive, integrated pain management programs and directives [11–13]. Recent surveys have found growing adoption of NPTs in healthcare systems [14–16], including the US Military Health System (MHS), in which 83% of military treatment facilities (MTFs) offer at least one type of NPT [17, 18]. However, in both civilian and military systems, utilization of NPTs falls short of the potential need and varies between healthcare facilities [19, 20]. Multiple studies have identified the underuse of NPTs [21, 22], guideline-discordant pain care [23, 24], and NPT access disparities [25].

Chronic pain conditions occur with regular frequency in service members, with the most recent data from 2014 indicating an incidence rate of 108 per 10,000 service member-years [26]. Moreover, the prevalence of chronic pain has been increasing in veterans newly being served, and such pain will likely become more common as these veterans age [27]. To better manage pain in service members, the MHS incorporated NPTs into clinical practice guidelines [13, 28] and established interdisciplinary pain management centers to deliver multimodal pain interventions for patients in some MTFs [29]. Currently, the Defense Health Agency is continuing to build policies and programs (e.g., Stepped Care Model for Pain) to implement NPT for pain management in the MHS [28–30]. This includes increasing the number of providers trained in auricular and medical acupuncture [31] and incorporating multidisciplinary pain management teams into patient-centered medical homes to promote care coordination, continuity, and access [29, 32].

While there are surveys that indicate many NPT approaches are being adopted by Military Treatment Facilities (MTFs), these surveys have not provided comprehensive estimates of system-wide capacity of professionals to deliver these therapies, nor examined the impact of increased capacity on access or utilization trends. Additionally, while one study in the Veterans Healthcare Administration demonstrated an association between facility-level NPT utilization and reduced initiation of long-term opioid therapy [20], there is a lack of research on whether changes in professional capacity in healthcare systems (e.g., number of chiropractors), including the MHS, have been sufficient to improve access and utilization of NPT. The objectives of the present study were (a) to measure professional capacity to deliver NPTs at MTFs (specifically, the mean number of available full-time equivalents (FTEs) per year per 100,000 patients for the occupations of physical therapist, chiropractor, and behavioral health provider) and (b) to test the associations between these professional capacity measures and measures of NPT utilization at the MTFs. The present study encompasses a time period during which the MHS paid significant policy and clinical attention to expanding the use of NPT as first-line intervention for pain. Thus, we anticipated significant expansions in both professional capacity and NPT utilization, and significant association between capacity and utilization between MTFs and over time. We also tested the associations of professional capacity at MTFs with service members’ utilization of NPT provided in the community to determine if NPT utilization increases at MTFs were offset by decreases in the community sector.

## Methods

### Data sources and sample

As part of the Substance Use and Psychological Injury Combat (SUPIC) study [33, 34], longitudinal information from multiple Department of Defense data sources was collected on all Army active duty and activated National Guard and Reserve component soldiers returning from deployment to Iraq or Afghanistan during the period from October 2007 to September 2014 (*N* = 863,855). All Reservists and National Guard members included in our cohort were activated for a deployment and covered by pre-mobilization or post-mobilization benefits or Transitional Assistance Management Program [35]. Sources for the ambulatory service utilization by these soldiers included both the Comprehensive Ambulatory/Professional Encounter Record (CAPER) database for direct care (care provided in the MTF) and the TRICARE Encounter Data -Non-Institutional (TED NI) database for purchased care (care provided in the community and reimbursed by TRICARE). Measures of clinical provider capacity for all MTFs in the US and abroad were derived from the Defense Medical Human Resources System Internet (DMHRSi). Observations from all data systems were created for the study period of October 1, 2009 to September 30, 2017.

### Definitions and measures

#### Measures of professional capacity to deliver NPTs at MTFs

The professional occupations measured included physical therapist, chiropractor, and behavioral health provider. We defined physical therapist as physical therapist and physiatrist; and behavioral health provider as psychiatrist, advanced practice nurse in psychiatry, nurse practitioner in mental health psychiatry, psychologists, clinical social worker, social worker, and behavioral sciences worker. For providers in these three professional groups, we used monthly assigned clinical hours at each MTF and corresponding patients served to calculate the MTF’s annual mean full-time equivalents (FTEs) and total number of patients served. The ratio of these two summaries created a standardized number of FTEs per 100,000 patients for each year.

#### Measures of NPT utilization outcomes among sample members

To examine NPT utilization rates, the soldiers that comprised the sample (hereafter referred to as sample members) were assigned each year to the MTF based on where they received the majority of their CAPER-recorded healthcare. Sample members’ utilization of NPTs was classified into modalities using procedure codes on healthcare records following a SUPIC algorithm previously published [34] (see listing in Additional file 1: Appendix 1). Five NPTs with sufficient utilization for individual analysis were: therapeutic exercise, chiropractic/spinal manipulation, acupuncture, massage, and transcutaneous electrical nerve stimulation (TENS). In addition, other NPT modalities were combined into two bundled NPT categories: other physical (including superficial heat, other physical therapy, traction, ultrasonography, lumbar support, cold laser), and psychoeducational (education self-care/management, biofeedback, health behavior interventions, stress-management, animal therapy, hypnotherapy, and art/music therapy). Based on these categories, we constructed total NPT initiators (users) and total encounters as measures of utilization for the seven NPTs per MTF per year for direct care and purchased care, leading to 28 utilization summary outcomes. These MTF utilization summaries were standardized as NPT initiators per 1000 sample members, and mean number of encounters per NPT user.

### Analysis

To begin, we presented descriptive statistics on professional capacity and NPT utilization over the study period. Following this, we used generalized estimating equation (GEE) models for each utilization outcome (with observations clustered within 130 MTFs) to test for associations between each FTE capacity measure and each utilization outcome. Additional adjustors in these models included type of MTF (clinic versus hospital), MTF geographic region (four TRICARE regions, Europe, and Asia), and MTF Service (Army vs other). Coefficients from the GEE models were interpreted as estimates of the change in NPT users per 1000 sample members (or change in average encounters per NPT user) due to a unit change in the capacity measure (i.e., FTE per 100,000 MTF patients). GEE models were conducted for 24 of our 28 utilization measures. Purchased care models for volume of acupuncture and chiropractic/spinal manipulation services were not performed because of insufficient numbers of users.

## Results

Our study sample comprised 863,855 soldiers, with an average of 5.34 years of care observed per soldier from 2008 to 2017, and 61.2 million healthcare encounter and claim records in direct (MTF-based) and purchased care (civilian) settings. Years of care were summed for each MTF based on the number of sample members with direct care records during the year. We merged NPT utilization measures with facility-level professional capacity measures for 10 years on 130 MTFs (1300 MTF-years) with complete data. Another 40 MTFs could not be included in the analyses because of incomplete data, often because the facility closed during the study period. These non-matched facilities were small special units which, while open, provided only a small proportion of all encounters for the study sample (see Study Flow, Additional file 1: Appendix 2). Of the total sample, NPT utilization at MTFs (direct care) was observed for 586,776 sample members (68%). A total of 93,304 sample members (11%) received at least some of their NPT in purchased care. At studied MTFs, we observed 348,214 encounters for therapeutic exercise, 163,461 for chiropractic/spinal manipulation, 159,433 massage, 118,299 TENS, and 28,375 acupuncture (Table 1). Prior studies have described the demographic and military service-related characteristics of the sample [34]. Fifty-four percent of MTFs were operated by the Air Force, 26% by the Army, and 21% by the Navy. Fifty-seven percent of MTFs were clinics without hospitals and 16% were located overseas.

**Table 1:** Military Treatment Facility (MTF) average total volume, sample size, and full-time equivalent (FTE) capacity measures (*n* = 130 facilities)

**MTF volume**	**2008**			**2013**			**2017**		
	**Mean**	**SE**		**Mean**	**SE**		**Mean**	**SE**	
All patients									
Persons Served	37,734	3,398.85		40,213	3,774.81		38,598	3,707.55	
Annual total encounters	197,546	20,399.12		231,583	25,232.14		227,352	25,357.20	
Study Sample									
Total sample persons	3,155	675.62		3,916	771.37		2,295	399.02	
Annual total encounters	25,220	5,433.10		50,521	9,854.55		32,957	5,941.18	
**Occupation**	**2008**			**2013**			**2017**		
	**Mean FTE**	**SE**	**# MTFs with**	**Mean FTE**	**SE**	**# MTFs with**	**Mean FTE**	**SE**	**# MTFs with**
Physical Therapist^a^	94.11	4.85	124	102.83	4.78	125	128.91	4.78	126
Chiropractor^a^	4.28	1.16	20	7.98	1.34	47	8.10	1.23	47
Behavioral Health Provider^a^	338.63	13.80	130	463.49	16.69	130	599.77	21.26	130

All years represent the period of a fiscal year, which begins October 1 of the prior calendar year and ends on September 30th of the stated year. The year 2013 was chosen as a mid-point in the data range to provide additional description of changes over the study period. All results are reported for only the 130 MTFs included in the study

^a^FTE is full-time equivalent positions assigned per 100,000 total persons served at MTF

### Trends in NPT utilization and MTF occupation capacity

The average number of total patients (service members, retirees, and family members) receiving care per MTF ranged from 37,734 in 2008 to 38,598 in 2017 (Table 1). The mean number of sample members served per MTF was 3,155 (SE = 675) in 2008 and declined to 2,295 (SE = 399) in 2017. This decline reflects the design of our study; we added sample members only until September 30, 2014 even though we examined their NPT utilization through fiscal year 2017. Notably, the number of encounters for the sample increased over time, even though the number of sample members in later years declined.

**Fig. 1:**
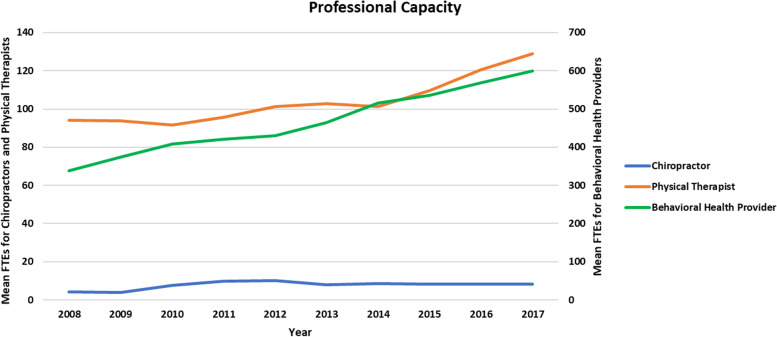
Average number of FTEs per 100,000 total patients at MTFs for three occupations (*n* = 1300 MTF-years)

Table 1 also presents average professional capacity per MTF for each of three occupations for the years 2008, 2013, and 2017. Figure 1 displays average capacity for occupations per MTF for each year within the period.

Professional capacity increased from 2008 and 2017 for each occupation. In 2008 nearly all MTFs had some physical therapist and behavioral health provider capacity, but only 15% of MTFs had even a part-time chiropractor. The number of MTFs with chiropractors increased the most (up 21 percentage points) from 2008 to 2017. Across MTFs, FTEs increased by 89% for chiropractors, 77% for behavioral health providers, and 37% for physical therapists from 2008 to 2017. By the end of 2017, the capacity of behavioral health providers were more than 3 times the capacity of physical therapists, the next highest profession. While chiropractic capacity nearly doubled from 2008 to 2017, compared to physical therapist capacity (129 per 100,000 patients in 2017) it remained relatively limited (8 per 100,000 patients in 2017).

**Fig. 2:**
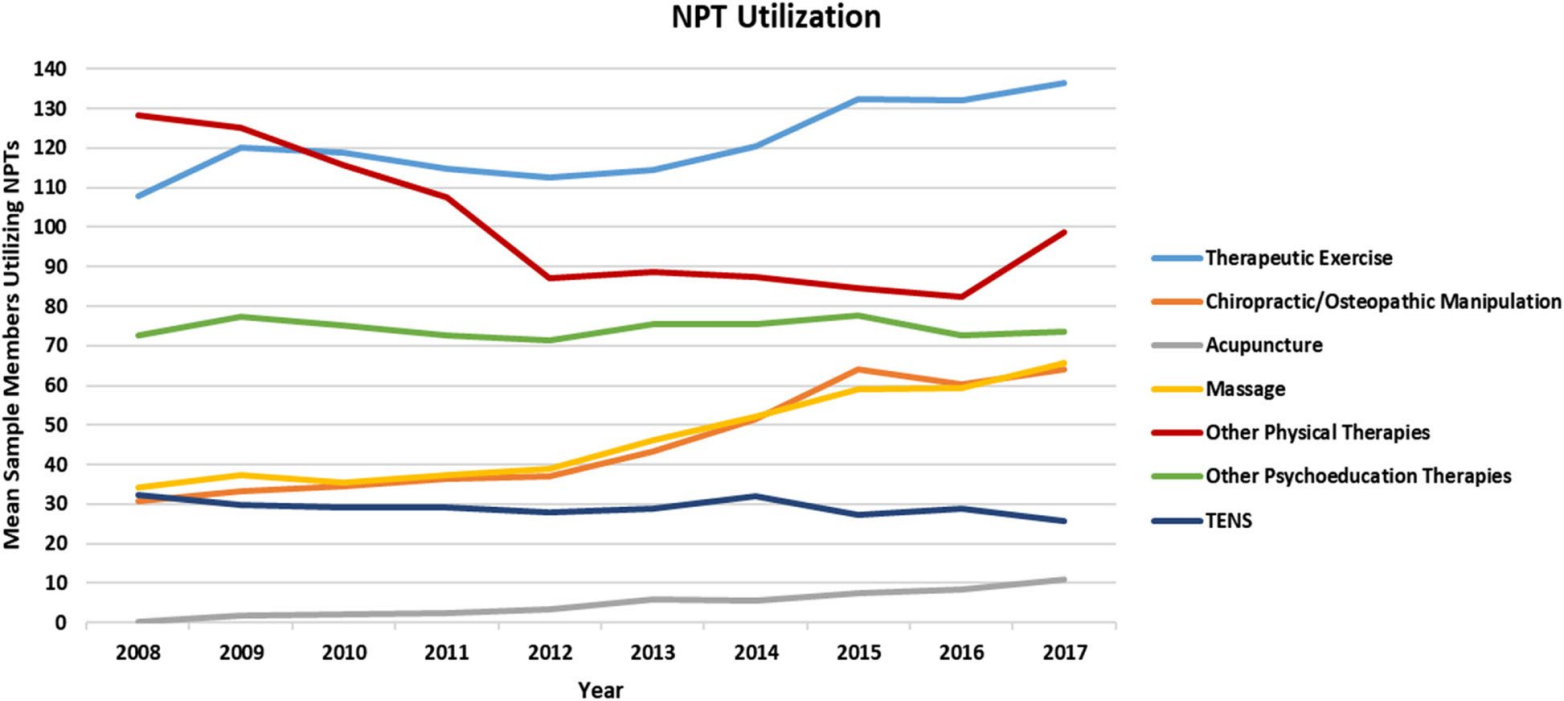
Average number of NPT users per 1000 sample members: October 2008 – September 2017 (*n* = 1300 MTF-years)

Changes in our outcome measures of NPT users at MTFs were not uniform (Additional file 1: Appendix 3; Fig. 2). While the greatest increase was observed in utilization of acupuncture (30 times more utilization in 2017 than 2008), utilization remained low; in 2017, the mean across MTFs was 11 users per 1000 sample members. From 2008 to 2017, utilization of chiropractic, massage, and therapeutic exercise increased 110%, 90%, and 30%, respectively. However, utilization of TENS and other physical therapies decreased by 23% and 21% respectively, and utilization of psychoeducation therapies remained flat in the study sample from 2008 to 2017. Consistent with the high availability of physical therapists, therapeutic exercise had the highest utilization rate in 2017 with a mean of 136.34 users per 1000 sample members.

Four NPTs experienced increases in the mean number of encounters per NPT user or utilization intensity. The greatest increase was observed for acupuncture, which increased 5.4 times. Mean encounters per MTF increased 1.42, 1.28, and 1.95 times for therapeutic exercise, chiropractic, and other psychoeducational therapy, respectively, but was relatively stable for massage and other physical therapies. Mean encounters per MTF for TENS decreased to 0.81 times the level seen in 2008.

### Association of Professional Capacity with NPT utilization in the MTFs

In Table 2, we present for each NPT the estimated impact of changes in occupation FTE (per 100,000 patients) on our utilization outcome measures based on regression model estimates. Greater physical therapist and chiropractor capacity were associated with significant increases in six out of the seven NPTs. For example, one FTE increase in physical therapist capacity was associated with an additional 0.61 user of therapeutic exercise per 1000 sample members, 0.33 user of physical therapy, 0.21 user of chiropractic services, 0.31 user of massage, 0.11 user of TENS, and 0.03 user of acupuncture. One FTE (per 100,000 patients) increase in chiropractic capacity was associated with an additional .94 user of chiropractic services per 1000 sample members, and other increases as well (Table 2). In terms of therapy modality, acupuncture initiators per 1000 sample members was significantly associated with increases in each occupation. Users of TENS and other physical therapy decreased by small amounts as behavioral health provider capacity increased.

**Table 2:** Association of full-time equivalent (FTE) capacity measures with nonpharmacologic therapy utilization in the direct care system: Results from fourteen multivariate regression models (*n* = 1300 observations)

	**Regression Coefficient Estimate (Standard Error)**^**a**^						
**Independent variable:**^**b**^	**Regression Model Dependent Variable: Users per 1000 Sample Members**						
	**Model 1** **TE**	**Model 2** **CH/OM**	**Model 3** **ACU**	**Model 4** **Massage**	**Model 5** **TENS**	**Model 6** **Oth PT**	**Model 7** **Oth PsychE**
Physical Therapist FTE	0.6056 (.0901)***	0.2058 (.0526)***	0.0341 (.0085)***	0.3109 (.0437)***	0.1187 (.0263)***	0.3287 (.0652)***	−0.0133 (.0889)
Chiropractor FTE	0.6506 (.2694)*	0.9363 (.1796)***	0.0682 (.0289)*	0.4599 (.1733)**	0.4586 (.1129)***	0.6905 (.2236)**	0.3487 (.3130)
Behavioral Health Provider FTE	−0.0266 (.0186)	−0.0072 (.0120)	0.0057 (.0021)**	−0.0092 (.0082)	− 0.0148 (.0065)*	−0.0330 (.0133)**	0.0514 (.0386)
**Independent variable:**^**b**^	**Regression Model Dependent Variable: Encounters per therapy user**^**c**^						
	**Model 1** **TE**	**Model 2** **CH/OM**	**Model 3** **ACU**	**Model 4** **Massage**	**Model 5** **TENS**	**Model 6** **Oth PT**	**Model 7** **Oth PsychE**
Physical Therapist FTE	0.0597 (.0186)**	0.0021 (.0051)	0.0104 (.0047)*	0.0121 (.0035)***	0.0057 (.0026)*	0.0109 (.0048)*	−0.0011 (.0064)
Chiropractor FTE	0.0911 (.0294)**	0.1013 (.0155)***	0.0308 (.0210)	0.0029 (.0079)	0.0231 (.0092)**	0.0327 (.0097)**	−0.0160 (.0116)
Behavioral Health Provider FTE	0.0051 (.0033)	−0.0007 (.0011)	0.0035 (.0015)*	−0.0013 (.0007)	−0.0018 (.0005)***	− 0.0031 (.0009)***	−0.0048 (.0013)***

*Abbreviations*: *ACU* Acupuncture, *CH/OM* Chiropractic and Osteopathic Manipulation, *Oth PsychE* Psychoeducation (see Additional file 1 for detailed list), *OthPTs* Other Physical Therapy (see Additional file 1 for detailed list), *TE* Therapeutic Exercise, *TENS* Transcutaneous Electrical Nerve Stimulation

*p ≤ 0.05; ** *p* ≤ 0.01; ***p ≤ 0.001

^a^Each regression model also included facility type (clinic vs hospital), facility branch (Army vs other) and 6 regions and patients were clustered by MTF

^b^Annual measure computed as FTE per 100,000 patients at the MTF

^c^For facilities with no utilization the number of visits per user was imputed as zero

Changes in professional capacity were also associated with significant changes in the intensity (number of encounters per user) of service utilization for many NPTs. Increases in physical therapist and chiropractor capacity was associated with increases in encounters per user of multiple NPTs. The largest effect observed was that a one FTE per 100,000 patients increase in chiropractic capacity was associated with an additional 0.10 encounters of chiropractic/spinal manipulation service per user.

### Association of professional capacity with NPT utilization in purchased care

We hypothesized that increased capacity at MTFs would not be associated with NPT utilization in our sample in purchased care. Economic theory might suggest a substitution of direct care for purchased care, once MTF occupational capacity is increased. However, we hypothesized that the unmet need for NPT was substantial and that substitution would be minimal. In Table 3 we present the results of ten regression models for purchased care. We found only a few significant associations between MTF occupational capacity and purchased care utilization outcomes. Increased chiropractor capacity was associated with increased purchased care users per 1000 for TENS, other physical therapy (e.g., heat therapy), and psychoeducation therapies. An increase in behavioral health provider FTEs was associated with a decrease in purchased care users of other physical therapy. Regarding purchased care NPT visits per user, our models indicated that chiropractor capacity was associated with increased therapeutic exercise, TENS, and other physical therapy visits per purchased care user. Physical therapist capacity was also associated with increased TENS purchased care visits per user. Only behavioral health capacity was negatively associated with the number of therapeutic exercise visits per user.

**Table 3:** Association of full-time equivalent (FTE) capacity measures with nonpharmacologic therapy utilization in the purchased care system: Results from ten multivariate regression models (*n* = 1300 observations)

**Independent variable:**^**b**^	**Regression Coefficient Estimate (Standard Error)**^**a**^				
	**Regression Model Dependent Variable: Users per 1000 sample soldiers**				
	**Model 1** **TE**	**Model 4** **Massage**	**Model 5** **TENS**	**Model 6** **Oth PT**	**Model 7** **Oth PsychE**
Physical Therapist FTE	0.0047 (.0230)	0.0114 (.0170)	0.0302 (.0177)	−0.0055 (.0103)	−0.0018 (.0029)
Chiropractor FTE	0.1239 (.0889)	0.0842 (.0640)	0.1573 (0.0806)*	0.1003 (.0483)*	0.0273 (.0124)*
Behavioral Health Provider FTE	−0.0077 (.0071)	−0.0063 (.0050)	− 0.0038 (.0051)	−0.0056 (.0029)*	− 0.0002 (.0010)
**Independent variable:**^**b**^	**Regression Model Dependent Variable: Encounters per therapy user**^**c**^				
	**Model 1** **TE**	**Model 4** **Massage**	**Model 5** **TENS**	**Model 6** **Oth PT**	**Model** **7 Oth PsychE**
Physical Therapist FTE	0.0226 (.0170)	0.0061 (.0060)	0.0116 (.0060)*	0.0014 (.0046)	0.0030 (.0077)
Chiropractor FTE	0.2251 (.0812)**	0.1052 (.0588)	0.0517 (.0248)*	0.0847 (.0284)**	0.0403 (.0490)
Behavioral Health Provider FTE	−0.0097 (.0046)*	−0.0023 (.0060)	−0.0016 (.0016)	− 0.0016 (.0013)	0.0018 (.0019)

Models 2 (CH/OM) and 3 (ACU) had insufficient observations to support analysis and are excluded from this table

*Abbreviations*: *ACU* Acupuncture, *CH/OM* Chiropractic and Osteopathic Manipulation, *Oth PsychE* Psychoeducation (see Additional file 1 for detailed list), *OthPTs* Other Physical Therapy (see Additional file 1 for detailed list), *TE* Therapeutic Exercise, *TENS* Transcutaneous Electrical Nerve Stimulation

*p ≤ 0.05; ** *p* ≤ 0.01; ***p ≤ 0.001

^a^Each regression model also included facility type (clinic vs hospital), facility branch (Army vs other) and 6 regions

^b^Annual measure computed as FTE per 100,000 patients at the MTF

^c^For facilities with no utilization the number of visits per user was imputed as zero

## Discussion

Over the last decade, the MHS has been reorganizing care to provide access to more NPTs in accordance with policy initiatives and clinical practice guidelines. This study found that from October 2008 to September 2017, the number of MTFs having capacity of chiropractors increased 21 percentage points, while the availability at MTFs of physical therapist and behavioral health providers was nearly universal at the start of the period. However, at the end of the period, still less than one-half of MTFs in our study had chiropractor FTEs. In addition to this diffusion of professional capacity to deliver NPTs to more MTFs, we found that the number of FTEs per 100,000 patients at MTFs increased for all studied occupations, most dramatically the concentration of chiropractors, although this occupation remained relatively less common than physical therapy and behavioral health. We also found that use of NPT services changed over the study period, with increases observed in therapeutic exercise, chiropractic care, massage, and acupuncture. Yet, these only modest increases in both professional capacity and NPT use observed during the same decade that the MHS was actively prioritizing NPTs suggest additional barriers may exist to effectively implementing NPTs in the MHS. Finally, observed decreases in TENS and other physical therapy interventions (such as ultrasound) may reflect a shift in clinical guidelines away from the use of passive therapies that occurred during our study period following Chou and Huffman’s 2007 [36] review of the evidence supporting NPT use for pain [10, 37].

Consistent with our hypotheses, increased professional capacity within MTFs was associated with increases in NPT utilization in MTFs among our sample members. However, capacity increases were not uniformly associated with increased utilization in all categories. Specifically, increased chiropractor and physical therapist capacity had the most widespread impact on the number of sample members receiving NPT care. When considering the magnitude of these associations, the present study examined NPT utilization in a relatively small sample compared to the total TRICARE population served by these facilities. Thus, while we have confidence in the direction and significance of our estimates of associations between professional capacity and NPT utilization, we realize that the magnitudes of these estimates are specific to this study. In other words, the estimates from this study may not be replicated in a study that included utilization data on all individuals receiving care at an MTF (e.g., retirees, family members, or service members who were never deployed).

We explored the effect of increasing chiropractic, physical therapy, and behavioral health professional capacity at MTFs on purchased care NPT utilization in our study sample. We found that capacity increases for chiropractic services within MTFs were associated with expanded use of several NPTs in community settings, suggesting that demand for care continued to outstrip supply available in MTFs. In part, the unmet need for NPT identified in the present study and lack of substitution of direct care for purchased care NPT may be indicative of overall lack of structured care algorithms and structured education to providers - in addition to the lack of NPT capacity. In mid-2018, the Defense Health Agency released a policy for pain management pathways [30], to include implementation of the Stepped Care Model for Pain. In the Stepped Care Model for Pain, providers are to refer patients for NPT, such as physical therapy and behavioral health, based on presenting pain severity and duration. In the event the local MTF does not have the capacity or availability to provide such care, patients can be referred to the network. It is unclear how these study results will change in the years following the Stepped Care Model for Pain implementation. Additionally, the lack of substitution of direct for purchased care NPT may be due to the lack of NPT providers overall. Evidence indicates that provider shortages across several NPT fields (e.g., physical therapy, chiropractic care), which may make it difficult for patients to engage in NPT, especially patients who are located at military bases in rural and provider shortage areas [38–40].

As integrative NPT providers become increasingly available and salient in the MHS, utilization may continue to increase as primary care and other providers may be more willing and have more experience recommending utilization of NPT. The number of NPT visits per NPT user also increased in association with increases in physical therapists and chiropractors. In this study, increases in behavioral health provider capacity at MTFs was common and in general was associated with a decrease in NPT utilization in the direct care system. We speculate that the behavioral health provider may provide an alternative approach to pain management, for example, providing guidance to the patient to re-conceptualize their pain or encouraging at home activities to address pain (e.g., meditation), thus reducing demand for physical therapies of pain. Future research should examine the role of behavioral health therapies among patients with pain conditions.

As the first study of occupational capacity and NPT utilization in a restricted military sample, we recommend that future studies examine samples of all service members to see if our results are replicated. We also recommend that future studies include additional occupational categories such as pain clinic providers who are being used in the MHS to address pain and mitigate opioid risks. Our team is continuing to investigate the utilization of NPT, and we intend to examine in future studies whether professional capacity and NPT utilization is associated with patient outcomes such as occupational functioning reduced opioid medication utilization.

### Limitations

This study had several limitations. First, our measures of capacity are likely to be underestimates because we limited our investigation to three occupations and we had no basis to estimate the amount of NPT services provided by other professionals (e.g., physicians trained on auricular acupuncture). Further, we measured provider capacity; yet some NPT services may also be offered by paraprofessionals not included in this study. While the number of FTEs may therefore be underestimated, our estimates of the presence or absence of a professional group are probably not affected since paraprofessionals would not be present in facilities without supervising providers. Second, our measures of specialty providers at MTFs relied upon reported FTE allocation in DMHRSi, and the accuracy of these data may vary by MTF. Further, because our data relied on procedure codes captured in CAPER, our measures of utilization likely underestimate services received inside MTFs as providers or paraprofessionals may not accurately code encounters or use the procedure codes we identified when they perform these services. A survey by Herman et al. [18] found that nearly a third of MTFs were uncertain about how consistently NPT was documented in the medical record, with only chiropractic and acupuncture documented consistently at 50% of MTFs. Further, Herman et al. indicated that documentation may only be captured narratively in clinical notes due to lack of Current Procedural Terminology (CPT) codes for specific types of complementary therapies. Due to these limitations of accurate services and FTE capture in CAPER and DMHSRi, our results must be interpreted with caution. Third, while we describe capacity of behavioral health providers as part of NPT service delivery, we do not know to what extent they are engaged in pain management care vs other kinds of intervention; thus, this capacity measure is clearly an over count. Fourth, the study uses a cross-sectional design, and thus we cannot interpret any associations as causal. Finally, this study is limited to observations on a specific restricted sample of service members, namely Army soldiers returning from a deployment during the study window. Therefore, findings may not be generalizable to samples of all military personnel. Specifically, our utilization measures are from a restricted sample, while the professional capacity measures are calculated using denominators for all patients at a facility. Thus, this study design may impair our ability to detect a relationship between capacity and utilization, and the magnitude of our estimates are specific to this study and do not generalize. Finally, we note that assessing services that sample members may have sought outside the MHS or purchased care system is beyond the scope of this study.

## Conclusions

We found increasing patterns of NPT utilization over the study period linked to growing professional capacity following MHS policy changes to improve access to NPTs. In addition to the direct relationship between capacity and utilization, increasing capacity may also have indirect effects on referring providers who serve as gatekeepers to NPT services by increasing NPT visibility in the healthcare system. However, the increases in overall NPT utilization were small in comparison to the high prevalence of chronic pain in military service member and Veteran populations [41, 42], and were not uniform across therapies. These findings suggest that additional factors influence utilization beyond supply and demand, and that policies to increase capacity alone may not be enough to ensure sustained guideline-concordant delivery of NPTs. Attention should also be given to other patient [43], provider [44, 45] and organizational-level incentives and constraints [46] that impact wide-scale adoption of NPTs as healthcare systems seek to expand utilization through policy initiatives and researchers seek to understand the effects of such initiatives on NPT uptake.

## Supplementary Information

**Additional file 1: A1.** Nonpharmacologic therapy (NPT) therapies and bundles with corresponding procedure codes. **A2.** Study Flow diagram. **A3.** Average nonpharmacologic therapy utilization at MTFs (*n* = 130 facilities).

## Data Availability

The Defense Health Agency’s Privacy and Civil Liberties Office provided access to Department of Defense (DoD) data. The datasets generated and analyzed during the current study are not publicly available according to our Data Sharing Agreement and are governed by the Defense Health Agency and Veterans Health Affairs.

## Abbreviations

FTE: Full-Time Equivalents
GEE: Generalized Estimating Equation
MHS: Military Health System
MTF: Military Treatment Facilities
NPT: Nonpharmacologic Therapy
SUPIC: Substance Use and Psychological Injury Combat study
TENS: Transcutaneous Electrical Nerve Stimulation
