# Supplementary material for: The relationship between capacity and utilization of nonpharmacologic therapies in the US Military Health System
Source: BMC Health Serv Res. 2022 Mar 7;22:312. doi: 10.1186/s12913-022-07700-4 (PMC8900315; doi:10.1186/s12913-022-07700-4)
Supplement: Supplementary file 1 — Additional file 1: A1. Nonpharmacologic therapy (NPT) therapies and bundles with corresponding procedure codes. A2. Study Flow diagram. A3. Average nonpharmacologic therapy utilization at MTFs (n = 130 facilities). [file 12913_2022_7700_MOESM1_ESM.docx]

**Additional file 1**

**A1.** *Nonpharmacologic therapy (NPT) therapies and bundles with corresponding procedure codes*

| **Therapy** | **Identifying codes** | **System** |
| --- | --- | --- |
| Therapeutic exercise | V6541  97110  97112  97113  97116  97150  97530  S9451  4245F  4242F | ICD-9 diagnosis  CPT  CPT  CPT  CPT  CPT  CPT  HCPCS  HCPCS  HCPCS |
| Spinal Manipulation or Chiropractic | 98925  98926  98927  98928  98929  S8990  S9090  98940  98941  98942  98943 | CPT  CPT  CPT  CPT  CPT  HCPCS  HCPCS  CPT  CPT  CPT  CPT |
| Acupuncture/Dry Needling | 97810  97811  97813  97814  S8930 | CPT  CPT  CPT  CPT  HCPCS |
| Massage | 97124  97140 | CPT  CPT |
| Transcutaneous Electrical Nerve Stimulation | 97014  97032  A4595  E0720  E0730  E0770  G0281  G0282  G0283  64550  A4556  A4557  A4558  E0731  0282T, 0283-0285T  L8680  L8681-L8688, L8689  E0762  E0745  0278T | CPT  CPT  HCPCS  HCPCS  HCPCS  HCPCS  HCPCS  HCPCS  HCPCS  CPT  HCPCS  HCPCS  HCPCS  HCPCS  HCPCS  HCPCS  HCPCS  HCPCS  HCPCS  HCPCS |
| Classified as Other Psychosocial Therapies | | |
| Biofeedback | 90875  90876  90901  96150 – 96115  E0746  97112 | CPT  CPT  CPT  CPT  HCPCS  CPT |
| Health behavior interventions | 96150 – 96115  96152  96153  96154  96155 | CPT  CPT  CPT  CPT  CPT |
| Self-management education | 97535  98960  98961  98962  99071  99078  S9445  S9446 | CPT  CPT  CPT  CPT  CPT  CPT  HCPCS  HCPCS |
| Stress Management | V6549_7  V6549_A  S9454 | ICD9 diagnosis  ICD9 diagnosis  HCPCS |
| Animal Therapy | S8940 | HCPCS |
| Hypnotherapy | 90880 | CPT |
| Art/Music Therapy | G0176  H2032 | HCPCS  HCPCS |
| Classified as Other Physical Therapies | | |
| Other Physical Therapy | V571  97016  97022  97034  G0151  97036 | ICD-9  CPT  CPT  CPT  HCPCS  CPT |
| Cold Laser | 97039 AND S8948  S8948 AND 97039 | CPT  HCPCS |
| Superficial Heat | 97010  97018  97024  97026  9335 | CPT  CPT  CPT  CPT  ICD9 |
| Ultrasonography | 97033  97035 | CPT  CPT |
| Traction | 97012  E0941  E0942 | CPT  HCPCS  HCPCS |
| Lumbar Support | L0621  L0623  L0625  L0626  L0627  L0628  L0630  L0631  L0633  L0634  L0637  L0638  L0972  L0976  L1005 | HCPCS  HCPCS  HCPCS  HCPCS  HCPCS  HCPCS  HCPCS  HCPCS  HCPCS  HCPCS  HCPCS  HCPCS  HCPCS  HCPCS  HCPCS |

^a^ ICD-9: International Classification of Diseases, ninth edition; CPT: Current Procedural Terminology; HCPCS: Healthcare Common Procedure Coding System codes.

^b^ We searched for Medical Nutrition Therapy and Christian Scientist Practitioner therapies but did not find utilization.

**A2.** Study Flow diagram


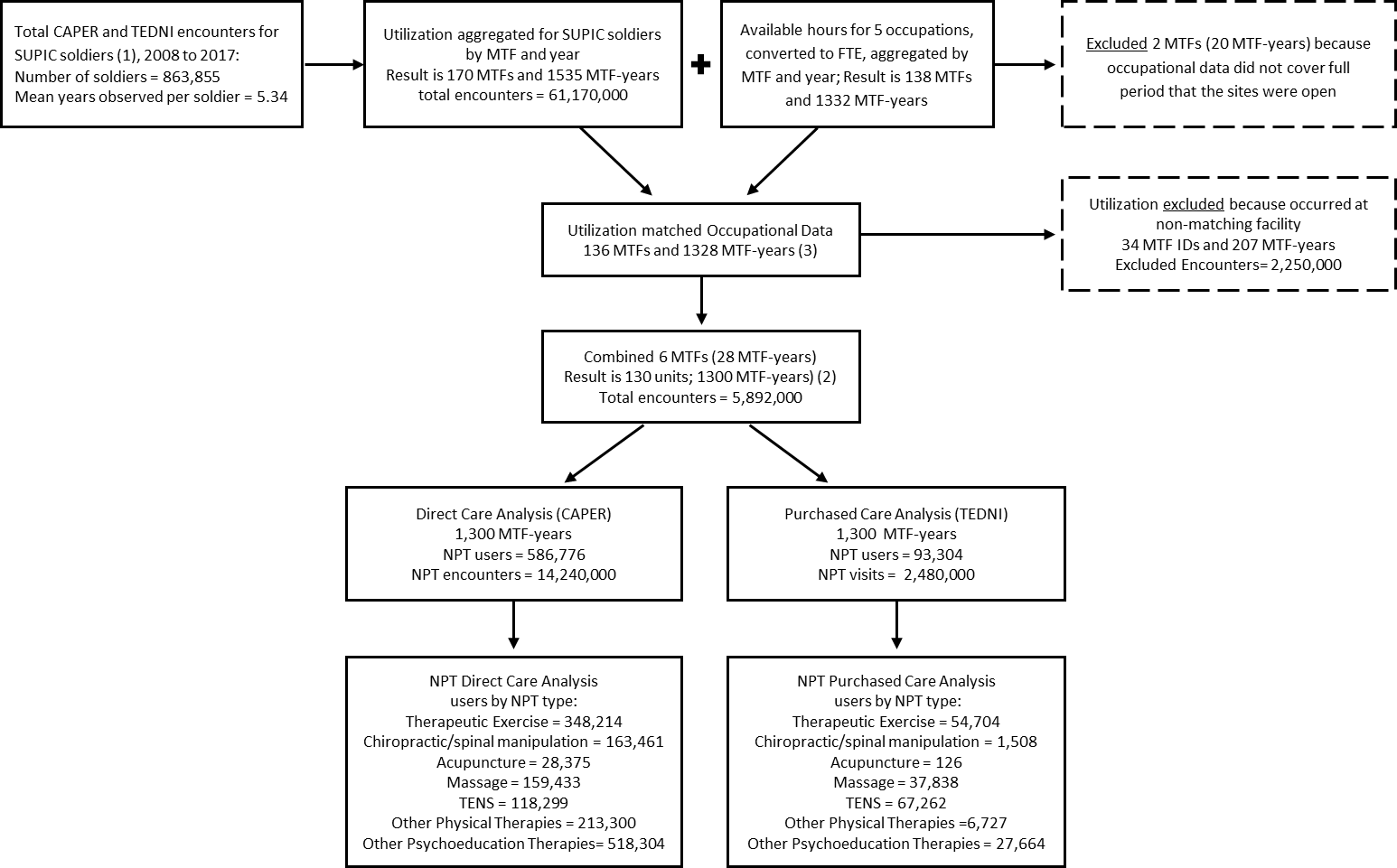


Notes: (1) CAPER are direct care encounter records and TEDNI are purchased care claims for the study window October 2008 to September 2017. SUPIC soldiers are all soldiers returning from an index deployment during the period October 2008 to September 2014. Utilization data in direct care and purchased care observed prior to and after deployment but not while deployed and receiving care for forward deployed medical groups.

(2) Visits and FTEs at 6 MTF sites which closed during study period were reclassified to the receiving MTF site

**A3.** *Average nonpharmacologic therapy* *utilization at MTFs (n=130 facilities)*

|  | **2008** | | **2013** | | **2017** | |
| --- | --- | --- | --- | --- | --- | --- |
|  | **Mean** | **SE** | **Mean** | **SE** | **Mean** | **SE** |
| Any NPT  Sample Members Served^a^  Encounters per NPT user^b^ | 231.17  13.80 | 16.30  1.26 | 276.16  19.00 | 14.20  0.79 | 336.22  18.27 | 13.84  0.62 |
| Therapeutic exercise  Sample Members Served^a^  Encounters per NPT user^b^ | 107.93  16.97 | 8.66  1.47 | 114.34  23.71 | 6.63  1.28 | 136.34  24.07 | 7.99  1.01 |
| Chiropractic/osteopathic manipulation  Sample Members Served^a^  Encounters per NPT user^b^ | 30.76  4.91 | 5.26  0.47 | 43.37  7.25 | 3.80  0.61 | 64.25  6.28 | 4.47  0.42 |
| Acupuncture  Sample Members Served^a^  Encounters per NPT user^b^ | 0.37  1.04 | 0.15  0.35 | 5.76  5.25 | 1.42  0.89 | 11.00  5.61 | 1.37  0.59 |
| Massage  Sample Members Served^a^  Encounters per NPT user^b^ | 34.20  3.97 | 4.42  0.37 | 46.22  5.62 | 3.55  0.59 | 65.70  4.16 | 4.56  0.22 |
| Transcutaneous Electrical Nerve Stimulation (TENS)  Sample Members Served^a^  Encounters per NPT user^b^ | 32.30  3.60 | 4.22  0.29 | 28.79  3.88 | 2.55  0.26 | 25.57  2.93 | 2.50  0.25 |
| Other Physical Therapies  Sample Members Served^a^  Encounters per NPT user^b^ | 72.56  5.98 | 6.27  0.39 | 75.29  7.38 | 4.77  0.40 | 73.53  6.05 | 5.54  0.33 |
| Psychosocial Interventions  Sample Members Served^a^  Encounters per NPT user^b^ | 128.31  4.50 | 15.11  0.47 | 88.76  7.59 | 11.01  0.60 | 98.68  8.76 | 9.97  0.47 |

Notes: All years represent the period of a fiscal year, which begins October 1 of the prior calendar year and ends on September 30^th^ of the stated year. The year 2013 was chosen as a mid-point in the data range. ^a^ Sample members served represents the number served per 1,000 sample members. ^b^Encounters per NPT user represents the number of encounters among sample members who used the NPT category.

**A4.** *Average nonpharmacologic therapy* *utilization in purchased care settings (n=130 MTF service areas).*

|  | **2008** | | **2013** | | **2017** | |
| --- | --- | --- | --- | --- | --- | --- |
|  | **Mean** | **SE** | **Mean** | **SE** | **Mean** | **SE** |
| Any NPT  Sample Members Served^a^  Encounters per NPT user^b^ | 10.22  7.69 | 2.81  1.24 | 27.58  12.28 | 3.23  1.21 | 35.16  10.74 | 3.72  0.82 |
| Therapeutic exercise  Sample Members Served^a^  Encounters per NPT user^b^ | 8.25  17.81 | 2.74  2.41 | 17.23  29.17 | 2.52  2.34 | 17.45  28.30 | 2.59  2.28 |
| Chiropractic/osteopathic manipulation  Sample Members Served^a^  Encounters per NPT user^b^ | 0.14  1.44 | 0.08  0.45 | 0.29  6.28 | 0.10  1.84 | 1.16  3.64 | 0.66  0.89 |
| Acupuncture  Sample Members Served^a^  Encounters per NPT user^b^ | ---  --- | ---  --- | 0.01  0.86 | 0.01  0.83 | ---  --- | ---  --- |
| Massage  Sample Members Served^a^  Encounters per NPT user^b^ | 4.34  2.75 | 0.99  0.39 | 11.34  4.94 | 1.95  0.49 | 12.79  5.13 | 2.06  0.45 |
| Transcutaneous Electrical Nerve Stimulation (TENS)  Sample Members Served^a^  Encounters per NPT user^b^ | 5.02  3.68 | 1.03  0.52 | 18.03  9.20 | 2.08  0.83 | 21.71  12.42 | 2.32  0.98 |
| Other Physical Therapies  Sample Members Served^a^  Encounters per NPT user^b^ | 3.40  3.06 | 0.76  0.43 | 8.35  6.45 | 1.23  0.85 | 5.43  5.86 | 0.89  0.74 |
| Psychosocial Interventions  Sample Members Served^a^  Encounters per NPT user^b^ | 0.65  2.78 | 0.36  1.72 | 1.53  4.04 | 0.33  1.16 | 2.22  6.58 | 0.61  2.95 |

Notes: All years represent the period of a fiscal year, which begins October 1 of the prior calendar year and ends on September 30^th^ of the stated year. The year 2013 was chosen as a mid-point in the data range. ^a^ Sample members served represents the number served per 1,000 sample members. ^b^Encounters per NPT user represents the number of encounters among sample members who used the NPT category.
